# Molecular Diagnosis of Bacterial Meningitis in Ethiopia: A Narrative Review of Current Evidence and Implementation Gaps

**DOI:** 10.1155/cjid/4447180

**Published:** 2026-08-10

**Authors:** Lema Ayele Tenkolu, Fikadu Balcha

**Affiliations:** ^1^ Department of Medical Laboratory Science, College of Health Science, Arsi University, Asella, Ethiopia, arsiun.edu.et

**Keywords:** bacterial, diagnosis, Ethiopia, meningitis, PCR, resource-limited settings

## Abstract

**Background:**

Bacterial meningitis (BM) remains a significant public health concern in Ethiopia, particularly due to diagnostic challenges posed by limited laboratory infrastructure, late patient presentation, and technical skills required for cerebrospinal fluid (CSF) sampling. Molecular diagnostic methods offer rapid, sensitive, and specific alternatives to conventional culture.

**Objective:**

This narrative review synthesizes available evidence on molecular diagnostics for BM in Ethiopia, evaluates their performance compared to conventional methods, and identifies barriers to implementation.

**Methods:**

A structured literature search was conducted in PubMed, Google Scholar, Scopus, and Web of Science (August 1977–September 2024) using the terms: Meningitis OR Bacterial meningitis AND Diagnostics OR Molecular diagnostics, including PCR, CRISPR, next‐generation sequencing (NGS), and MALDI‐TOF. The review used a repeatable research selection procedure, well‐defined inclusion/exclusion criteria, and PRISMA reporting requirements. Studies from Ethiopia and other low‐resource settings were included. After screening titles/abstracts and full texts, 66 articles were selected.

**Results:**

In Ethiopia, multiplex PCR detected bacterial DNA in 10%–22% of the CSF samples, whereas culture was positive in only 0.5%–1% of the cases, primarily due to pre‐admission antibiotic use. Globally, molecular panels (e.g., BioFire FilmArray ME) show > 90% sensitivity and specificity. Advanced techniques (CRISPR‐Cas, NGS, and MALDI‐TOF) have not yet been implemented in Ethiopian routine diagnostics.

**Conclusion:**

Molecular methods vastly improve the detection of BM in Ethiopia, but high costs, infrastructure gaps, and shortage of trained personnel prevent widespread adoption. Molecular assays have outstanding analytical sensitivity, but culture remains the gold standard for confirmation of the viability of live pathogens. Implementation gaps require urgent targeted investment in point‐of‐care diagnostics and specialized transport networks.

## 1. Introduction

### 1.1. Background

Meningitis is a very serious disease of the central nervous system (CNS) that inflames the defensive membranes of the brain and spinal cord. This inflammation is the result of an infectious agent in the cerebrospinal fluid (CSF) surrounding the brain and spinal cord [1].

Bacterial meningitis (BM) continues to be a significant infectious disease globally, despite the progress made in treatment and vaccination [2]. It is a serious and life‐threatening condition that requires early diagnosis and treatment [3–5].

The incidence of BM and its mortality rate vary from one area to another, age group, and type of organism [6]. In areas where human immune deficiency (HIV) is widespread, such as sub‐Saharan Africa, the mortality rate could potentially rise to 50% [7, 8]. In Ethiopia, BM accounts for 6%–8% of hospital admissions and has a fatality rate ranging from 22% to 28% [9]. The nation is located in the “meningitis belt” of Africa, where seasonal epidemics are common, especially from December to June during the dry season. Although new serogroups like NmW and NmX have been discovered, the most common pathogens are *Neisseria meningitidis*, *Streptococcus pneumoniae*, and *Haemophilus influenzae* Type b (Hib) [10–12].

The most common clinical symptoms observed in patients with BM include high fever, headache, and loss of consciousness. Two‐thirds of adult patients with BM present the triad although all patients experience at least one of these symptoms [13]. The classic symptoms may not be observed in neonates and elderly patients [14].

Diagnosing of BM in low‐resource settings like Ethiopia is challenging due to late patient presentation [15], limited diagnostic facilities [16], and the high technical skills required to collect CSF samples (obtaining a CSF sample, particularly from young infants, is technically very difficult and invasive/painful for the patient). Conventional tests such as white blood cell count (WBC), glucose, proteins in CSF, and gram staining are inexpensive and fast but not very specific [17, 18]. Bacterial cultures require several days for a definitive diagnosis to be made; the need for the best possible environment as well as their sensitivity and specificity are low relative to the molecular method [19].

In conclusion, traditional diagnostic techniques lack specificity and are inadequate for prompt and precise diagnosis, despite being quick and economical. Higher sensitivity and specificity, which are critical in addressing the high morbidity and mortality linked to delayed diagnosis, are among the promised benefits of molecular techniques. However, the diagnosis process is still complicated by issues with preadmission antibiotic therapy, reagent prices, and the invasive aspect of CSF collection.

## 2. Materials and Methods

This narrative review was carried out in accordance with PRISMA reporting requirements. A comprehensive search was made on multiple database including, PubMed, Google Scholar, Scopus, and Web of Science for articles published from August 1977 to September 2024. Meningitis OR Bacterial meningitis AND Diagnostics OR Molecular diagnostics was the search term used. Research on the diagnosis of BM and molecular methods that satisfy the requirements of being published in English and subjected to peer review were included. Research on molecular diagnostic techniques for BM, studies from Ethiopia or other low‐resource environments, original studies, reviews, or case series, and full‐text availability were the inclusion criteria. The following criteria were used for exclusion: (1) titles unrelated to the current issue, (2) non‐peer‐reviewed papers, (3) studies that did not concentrate on the diagnosis of BM, and (4) works published before to 1977. Studies were also required to provide Ethiopia‐specific data or directly relevant comparisons for low‐resource settings. A total of 116 studies were retrieved using this search method. The titles and abstracts of these papers were used to determine their relevance. To ensure they aligned with the review’s goals, full‐text articles that satisfied the relevancy criteria were examined, and 66 papers were cited in this review. There were two steps in the study selection process: (1) the author screened the title and abstract and (2) the complete text was reviewed for eligibility. The selection process was monitored using a PRISMA flow diagram (available upon request). EndNote Version 21 was used to manage references.

## 3. Results and Discussion

### 3.1. Etiology of Meningitis With an Ethiopia Focus

The most frequent bacterial infections that cause meningitis in Ethiopia are Hib, *S. pneumoniae*, and *N. meningitidis*. However, current surveillance has detected rising serogroups such as NmW and NmX, with hypervirulent strains of *Klebsiella pneumoniae* [12, 20, 21].

Gram‐negative enteric rods such as *Esherichia coli* and some other organisms such as *Listeria monocytogenes* and *Streptococcus agalactiae* are more common during the neonatal period [22], while S*. pneumoniae*, *N. meningitidis*, and *H. influenzae* are more common in children and young adults [23].

#### 3.1.1. *N. meningitidis*

A Gram‐negative diplococcus is an obligate commensal bacteria residing in the human nasopharynx. The highest incidence of nasopharyngeal carriage of *N. meningitidis* is in adolescents, especially those residing in overcrowded spaces [24]. Particularly prone are school‐going children and college students, household contacts of meningococcal patients, and also military recruits [25]. *N. meningitidis* can exist with or without a polysaccharide capsule. However, nearly all meningococcal meningitis infections are caused by the capsulated form. Based on the polysaccharide characteristics, *N. meningitidis* can be divided into at least 12 different serogroups. Serogroups A, B, C, W, X, and Y are isolated in almost 90% of the infections patients. Serogroup A used to predominate in Ethiopia, but since the MenAfriVac vaccination was introduced in 2010, serogroups W and X have become serious public health risks [11, 12].

#### 3.1.2. *S. pneumoniae*

*S. pneumoniae* is a significant cause of BM. There are more than 90 known serotypes based on the polysaccharide capsule. The most common serotypes causing invasive pneumococcal disease worldwide include 1, 5, 6A, 6B, 14, 19A, 19F, and 23F [26]. No age group is exempt from pneumococcal meningitis. It usually affects small children under the age of 2 years. Immunocompromised people are also at a higher risk of acquiring pneumococcal meningitis. *S. pneumoniae* spreads through respiratory droplets, similar to *N. meningitidis* and *H. influenzae.* The high rates of pneumococcal infections may partly be due to the high carriage rates among the general population. Children under 6 years of age have the highest rates of nasopharyngeal carriage [27]. This high carriage rate among infants and young children explains why pneumococcal disease is the leading cause of vaccine‐preventable deaths in this age group [28]. Molecular research has confirmed the presence of pneumococcal meningitis in CSF samples from probable meningitis patients in Ethiopia, where the disease is still widespread [11, 29].

#### 3.1.3. Hib

The polysaccharide capsule forms the basis for classification into six serotypes—a, b, c, d, e, and f—of *H. influenzae*. Of these, the serotype b, commonly referred to as Hib, is associated with a high incidence of meningitis infections. Indeed, before the introduction of the vaccine, Hib was the leading cause of acute BM in children under the age of 5 years. The introduction of the Hib vaccine has since drastically reduced the incidences of this infection. Vaccination efforts have considerably reduced the diseases, thus showing that the vaccine is effective in maintaining protection for the populations at risk, notably children below 5 years of age [30]. Although the number of Hib illnesses in Ethiopia has decreased since the vaccination was introduced, instances may still go unreported due to a lack of diagnostic tools.

#### 3.1.4. *S. agalactiae*/Group B Streptococcus (GBS)

*S. agalactiae*, commonly known as GBS, is a Gram‐positive bacterium that is the leading cause of BM, particularly in neonates. *S. agalactiae* meningitis is more commonly associated with neonates; it can also occur in adults, particularly in those with chronic medical diseases or comorbidities, and even in healthy individuals [31, 32]. Study in Ethiopia found GBS by PCR in 64% of the suspected baby meningitis cases in Ethiopia, with no culture positivity, demonstrating the higher sensitivity of molecular techniques [33].

#### 3.1.5. *L. monocytogenes*

An intracellular facultative anaerobic bacterium that is Gram‐positive and can cause meningitis in several people, including the elderly, immune compromised, and neonates. It is infected in humans by eating contaminated foods, such as prepared foods, deli meats, and soft cheeses [34, 35]. It has 13 distinct serotypes, which differ in flagellar and depend on surface antigens, but almost three serotypes only (1/2a, 1/2b, and 4a) cause all human diseases [36]. Listeriosis should be taken into consideration in high‐risk groups, notwithstanding the paucity of evidence from Ethiopia.

#### 3.1.6. *E. Coli*

*E. coli* is the most common Gram‐negative bacillary organism that causes meningitis, particularly during the neonatal period or in babies under 3 months of age. *E. coli* strains possessing the K1 capsular polysaccharide are predominant (approximately 80%) among isolates from neonatal *E. coli* meningitis, and most of these K1 isolates are associated with a limited number of O serotypes [37]. The infection is a serious condition, especially in newborns and premature babies [38].

#### 3.1.7. *K. pneumoniae*

*K. pneumoniae* strains causing meningitis contain distinct resistance genes, capsular serotypes, virulence genes, and related clones, which might eventually lead to plasmid‐mediated resistance and virulence transmission, which can have a negative influence on patient prognosis [20]. Individuals with meningitis caused by hypervirulent carbapenem‐resistant *K. pneumoniae* (Hv‐CRKP) had higher mortality than the other patients. The high percentage and fatality of *K. pneumoniae*‐caused meningitis, particularly by Hv‐CRKP strains, should be of significant concern [21].

#### 3.1.8. Other Bacterial Etiologies

Other uncommon causes of BM include *Staphylococcus aureus, Pseudomonas aeruginosa,* and some *Enterococci*. They are usually associated with healthcare‐associated infections and may be acquired after trauma or some surgical interventions [39].

### 3.2. Epidemiology

BM has been a serious health concern since several years ago. *N. meningitidis*, Hib, and *S. pneumoniae* were the most common causes of BM between 1950 and 1970; they accounted for 70% of the cases of meningitis in the United States. In 1977, Centre for Disease Control and Prevention (CDC) national surveillance showed 3 cases per 100,000, with the highest case rate among children under one year old [1, 40].

Since the introduction of vaccination programs for both *N. meningitidis* and Hib, incidence rates have drastically declined. From 2.00 cases per 100,000 in 1998, the number of cases of BM declined to 1.38 cases per 100,000 in 2007. During this time, the median age of patients affected increased from 30.3 to 41.9 years [41]. According to the report from 2017, the number of cases of meningococcal disease had decreased, indicating the efficacy of the immunization campaign [13, 42].

In developed countries, the overall incidence of BM has stabilized. For example, in England, the mean annual incidence from 2012 to 2019 remained at 1.49 cases per 100,000, with variations in causative pathogens by age group. *S. agalactiae* meningitis was predominant among the infants [43] though that of pneumococcal meningitis increased among the older adults [10].

Meningitis is still endemic in Ethiopia, with the WHO reporting frequent outbreaks. Thirteen people died as a result of an outbreak that affected 11 districts in 2022 alone [12]. Peak transmission occurs during the dry season, which runs from December to June. Despite vaccination campaigns, *N. meningitidis* serogroups W and X have surfaced, along with pneumococcal and Hib infections [10, 11]. Many instances go undetected or are incorrectly identified due to limited laboratory capacity.

### 3.3. Clinical Presentation

According to the study, the classic triad of fever, neck stiffness, and a change in mental status was present in only 44% of the episodes; however, 95% had at least two of the four symptoms of headache, fever, neck stiffness, and altered mental status [12, 44]. Meningitis was difficult to diagnose since the whole triad of nuchal stiffness, fever, and disturbed mental state was rarely present [45]. Patients’ presentations change depending on their age. Older patients were more likely to experience focal neurologic impairments and disturbed mental state although they were less likely to experience headaches and neck stiffness [46]. The presentation varies in young children; in children, the signs and symptoms were often nonspecific, and they included hyper or hypotonia, poor feeding, and irritability [13].

### 3.4. Complications

BM can cause complications such as hearing defects, speech abnormalities, intellectual impairment, learning difficulties, and seizures [42]. Complications are more common in small children, thus causing serious neurological defects, which often tend to be long‐standing. These complications are local neurological deficits, paralysis of the limbs, developmental disabilities, seizures, cerebral abscesses, and hydrocephalus. Most of these complications in children usually resolve within 2‐3 years, but 10% of the children may develop complications that persist throughout their lives [47].

### 3.5. Laboratory Diagnosis

#### 3.5.1. Conventional Methods

BM is best diagnosed by combining clinical assessment with laboratory evidence of the causative organism. The presence of bacteria in the CSF forms the basis of the diagnosis. The CSF characteristics highly suggestive of BM include an elevated CSF cell count (> 500/μL), predominantly neutrophils, increased protein levels in the CSF (> 1 g/L), increased levels of CSF lactate (31.53 mg/dL), and a lowered CSF/blood ratio of glucose (< 0.4). Gram staining and culture are conventional approaches for identifying bacteria in CSF samples. However, the yield may be decreased if there is previous antibiotic treatment. The Gram stain and colony appearance can give hints to the specific organism [48] (Table 1).

**TABLE 1:** Classification of bacteria causing acute meningitis based on Gram‐reaction and colony appearance [49].

Bacterium	Gram‐reaction	Colony appearance
*A. Gram-positive bacteria*		
*S. aureus*	Gram‐positive cocci (clusters)	Yellow and round, opaque colonies may exhibit hemolysis on blood agar
*S. pneumoniae*	Gram‐positive diplococci (lanceolate, short chains)	Little, clear, dome‐shaped colonies with a “fried egg” look
*S. agalactiae*	Gram‐positive cocci (pairs or chains)	Small, grayish‐white colonies exhibits beta‐hemolysis on blood agar
*L. monocytogenes*	Gram‐positive bacilli (small rods)	Tiny, translucent, grayish‐white colonies; shows beta‐hemolysis on blood agar

*B. Gram-negative bacteria*		
*N. meningitidis*	Gram‐negative diplococci (“coffee beans”)	Small, greyish‐white colonies with a wet, slimy appearance
*H. influenzae*	Small Gram‐negative pleomorphic rods	Small, transparent, grayish‐white colonies; requires special growth factors (X and V factors).
*E. coli*	Gram‐negative rods (single or paired)	Large, lactose‐fermenting colonies that are pink or red on MacConkey agar.
*K. pneumoniae*	Gram‐negative bacilli (singles or pairs)	Large, mucoid colonies with a distinct “jelly” appearance due to capsule formation.

Culture diagnostics are hampered by massive systemic challenges in low‐resource settings. From a logistical point of view, there is a severe shortage of specialized transport media such as Trans‐Isolate Medium (TIM) apart from the widespread use of self‐medication of antibiotics prior to admission, which sterilizes clinical samples. This is a major diagnostic challenge in the management of BM in Ethiopia. Considering the long distances between the collection points for remote rural patients and centralized tertiary referral hospitals where functioning culture setups exist, fastidious pathogens often do not survive the transit period without proper transport stabilization resulting in false‐negative cultures.

#### 3.5.2. Molecular Methods

Nucleic acid amplification tests (NAATs) such as multiplex PCR testing can provide rapid and accurate diagnoses for BM [50, 51]. Depending on the method used, results can be available in as little as 15 min and no longer as 3 h for a single sample [52, 53]. The time to results is greatly increased by shipping if the PCR test is not conducted on‐site. Although the sensitivity and specificity of commercial NAATs are frequently greater than 90% for each bacterial pathogen, test performance differs depending on the pathogen and assay [51, 52, 54, 55]. For example, the BioFire Film Array meningitis/encephalitis panel (FilmArray ME) is a PCR panel that rapidly (1 h) detects 14 pathogens, including six bacteria, seven viruses, and one fungus, directly from CSF [53, 54].

For the detection of nucleic acids from CSF, sophisticated molecular methods including matrix‐assisted laser desorption ionization time‐of‐flight mass spectrometry (MALDI‐TOF MS), next‐generation sequencing (NGS), and clustered regularly interspaced short palindromic repeats (CRISPR–Cas) show promise [55–60]. However, because of the high price and infrastructural requirements, Ethiopian routine diagnostics have not yet adopted these technologies. MALDI‐TOF MS is included here as a high‐throughput method for identifying cultured pathogens, despite the fact that it identifies ribosomal proteins rather than nucleic acids. Although no Ethiopian studies have confirmed the use of CRISPR‐based diagnostics (such as SHERLOCK and DETECTR) for meningitis, they provide quick, paper‐strip readouts and could be modified for low‐resource situations. In a similar vein, NGS enables objective pathogen detection but is still unaffordable.

Multiplex‐PCR is a very useful tool for early diagnosis in BM. It not only provides the identification of infection but also important epidemiological data useful for vaccination programs to prevent transmission of diseases [29, 61] (Table 2).

**TABLE 2:** Summary of enhanced detection BM by molecular approach in clinical samples.

Studies	Years	Specimen	Approach	Remark	Reference
India	2022	CSF samples (*n* = 113)	Culture, multiplex PCR	Multiplex PCR detected 15% positive samples; culture detected only 10%.	[62]
United Kingdom	1996	Blood samples (*n* = 80)	PCR for meningococcal meningitis	PCR showed 100% sensitivity and specificity for diagnosed cases. PCR on blood is practical for patients where lumbar puncture is contraindicated.	[63]
India	2022	CSF samples (*n* = 178)	Real‐time PCR and culture	Real‐time PCR exhibited 100% sensitivity and 81.3% specificity for compared to culture.	[64]
Egypt	2022	CSF samples (*n* = 48)	Multiplex PCR	94% sensitivity and 100% specificity for diagnosing bacterial meningitis.	[65]
Ethiopia	2018	CSF samples (*n* = 218)	PCR and culture	21 (10%) of CSF samples were PCR‐positive; only 1 positive for culture.	[29]
Ethiopia	2019	CSF and serum samples (*n* = 23 pairs)	PCR technique	22 (96%) CSF samples had detectable NmDNA; 6 (26%) serum samples were positive for NmDNA.	[66]
Ethiopia	2020	CSF Samples (*n* = 72)	PCR technique and culture	*S. agalactiae* was detected in 64% of CSF samples by PCR with culture zero detection.	[33]

Molecular methods are very sensitive for detection of bacterial DNA but a positive PCR does not always mean an active, live infection. Disruption of the blood–brain barrier from other etiologies may introduce bacterial DNA into the CSF, or DNA may be derived from nonviable organisms after antibiotic therapy. Therefore, bacterial culture remains the gold standard for diagnosis of active infection as it detects viable, replicating bacteria. Molecular and culture methods should be considered complementary, not alternative.

## 4. Evaluation of Diagnostic Performance Across Studies

Significant variation in diagnostic performance can be seen when comparing the Ethiopian studies. In 218 CSF samples, conducted in Jimma, they found 10% PCR positivity compared to 0.5% culture positivity [29]. GBS was detected by PCR in 64% of the newborn CSF samples with no culture yield, according to another study [33] (Table 2). These results highlight the higher sensitivity of molecular techniques, especially when preadmission antibiotic use renders culture useless. Nevertheless, no Ethiopian data are available for CRISPR, NGS, or MALDI‐TOF, and none of these investigations used multiplex panels such as BioFire FilmArray. Small sample numbers, single‐center designs, and a lack of cost‐effectiveness analysis are some of the limitations.

## 5. Conclusion

The diagnosis of BM in low‐resource settings like Ethiopia presents significant challenges, including late presentations, limited diagnostic facilities, and the technical difficulties associated with CSF sample collection. Furthermore, the logistical nonavailability of specialized transportation media such as TIM seriously hampers regional culture capacity during long‐distance transit from rural settings. Ethiopian studies showing 10%–22% PCR positivity against < 1% culture positivity suggest that molecular techniques like multiplex PCR significantly increase detection rates as compared to culture. However, wider implementation is hampered by high costs, infrastructure deficiencies (such as inconsistent energy and a lack of PCR), and a lack of skilled workers. There is an urgent need for task‐sharing techniques like centralized reference laboratories with sample transport networks and focused investments in point‐of‐care molecular testing like GeneXpert. While conventional diagnostic methods are quick and cost‐effective, they lack specificity and are insufficient for timely and accurate diagnosis. Molecular methods offer promising advantages, including higher sensitivity and specificity, which are crucial in addressing the high morbidity and mortality associated with delayed diagnosis. However, due to the fact that molecular tools detect nucleic fragments that might pass through a compromised blood–brain barrier through nonspecific mechanisms, DNA detection cannot definitively establish active pathology.

Therefore, the results of this review underline the significance of traditional culture detection methods as the ultimate gold standard because of their unique ability to detect live, active infections. However, the challenges posed by cost and infrastructure continue to complicate the introduction of new technologies in resource setting like Ethiopia.

## Funding

No funding was received for this manuscript.

## Conflicts of Interest

The authors declare no conflicts of interest.

## Data Availability

The data that support the findings of this study are openly available in PubMed and Google Scholar at https://pubmed.ncbi.nlm.nih.gov/.
